# Post-mortem diagnosis of septic arthritis by Pasteurella multocida: a case report and literature review of fatal septic arthritis

**DOI:** 10.1007/s12024-024-00929-x

**Published:** 2025-01-11

**Authors:** Arianna Giorgetti, Simone Santelli, Ilenia Filipuzzi, Maria Paola Bonasoni, Giuseppe Basile, Filippo Pirani, Susi Pelotti

**Affiliations:** 1 Department of Medical and Surgical Sciences, Unit of Legal Medicine, University of Bologna, Via Irnerio 49, 40126 Bologna, Italy; 2 Pathology Unit, Azienda USL-IRCCS Di Reggio Emilia, Via Amendola 2, 42122 Reggio Emilia, Italy; 3 Trauma Unit and Emergency Department, IRCCS Galeazzi Orthopedics Institute, Milan, Italy

**Keywords:** Forensic pathology, Fatal septic arthritis, Autopsy, Post-mortem diagnosis of sepsis, Multidisciplinary approach

## Abstract

The diagnosis of septic arthritis remains challenging in the clinical setting, often leading to a suspicion for medical liability. Our purpose is to describe an unusual case of a post-mortem diagnosis of *P. multocida* fatal septic arthritis, in a healthy 67-year-old woman presenting with pain in the right shoulder. Moreover, a literature review of cases of fatal septic arthritis is provided. The multidisciplinary approach consisted of a forensic autopsy and additional post-mortem analyses (microbiology, biochemical analyses, histopathology, and revision of radiological images) carried out during the prosecutor’s investigation for medical liability. A systematic review of the literature was performed to collect cases of fatal septic arthritis and to understand its frequency and characteristics. No clear cause of death was determined after the autopsy, that only highlighted swelling and purulent exudation in the right glenohumeral joint. The microbiological swab performed on the shoulder tested positive for *P. multocida*, while histopathological and biochemical data were consistent with a sepsis. These results guided the interview with the woman’s relatives, until a history of a previous contact with a stray cat emerged. The cause of death was deemed as fatal septic arthritis caused by *P. multocida,* occurred after cat scratches and bites and only diagnosed post-mortem. The review of the literature provided 15 articles about fatal septic arthritis, only 1 caused by *P. multocida*, and all with ante-mortem diagnosis. Given the nonspecific symptoms, usually including a localized pain, and the absence of a clear history, e.g. of animal contact, septic arthritis might represent an under-reported clinical and pathological diagnosis, leading to a judicial autopsy for the suspicion for medical liability. The post-mortem examination, following a multidisciplinary approach including integration of the clinical history, microbiological and histopathological analysis, could represent the only opportunity for the diagnosis of the cause of death.

## Introduction

*Pasteurella (P.) multocida* is a gram-negative bacterium. It is a non-motile, facultative anaerobic, fermentative coccobacillus found in the oropharynx of healthy animals, particularly cats, dogs, and pigs, as well as various wild animals [1, 2]. Cats and dogs are the main reservoirs for human infection, with high carriage rates ranging from 70 to 90% in cats and from 20 to 50% in dogs, respectively [3–5]. Indeed, human infections due to *P. multocida* are closely associated with animal exposure and usually involve soft-tissue sites after animal bites or scratches [6, 7]. *Pasteurella* species (spp) are the most frequent isolates from dog bites and cat scratches and bites, accounting for 50% and 75% of cases, respectively [8–10]. The most common consequence of *P. multocida* infection in humans is local skin and soft tissue infection (e.g. cellulitis, abscesses or purulent wounds), although serious systemic diseases may occur (e.g., meningitis, empyema, pneumonia, peritonitis, osteoarticular infections, endocarditis, and septicemia), requiring urgent medical advice [2].

The forensic literature on fatal infections by *P. multocida* is scarce and mostly includes cases of septic shock following pneumonia or, more recently, infective aortic aneurysm [11, 12]. Although septic arthritis by *P. multocida* has been described in living patients, to the best of our knowledge there are no current reports about fatal septic arthritis caused by this or other pathogen detected at autopsy.

Herein, we present a case of fatal septic arthritis caused by *P. multocida*, likely occurred after cat scratches and bites, in which the microorganism was microbiologically isolated in post-mortem samples of the involved joint.

In addition, a literature review of fatal cases of septic arthritis is provided, in order to assess the prevalence of fatal septic arthritis by *P. multocida* and to evaluate similarities and differences with fatal septic arthritis caused by other pathogens.

## Case history

A 67-year-old woman presented to the emergency department (ED) with a complaint of right shoulder pain, that had started a few days earlier and had not responded to analgesic therapy. The patient’s past medical history was significant for degenerative polyarticular osteoarthritis, with no previous trauma reported except for a left wrist injury. The first examination was unremarkable aside from a rather undefined limitation of the right shoulder movements. X-rays showed severe arthritis of the right glenohumeral joint; blood tests were not performed. The patient was administered acetaminophen and discharged after few hours with a prescription of a shoulder ultrasound and a diagnosis of degenerative joint arthritis.

Two days after, the patient presented to the ED of a different hospital due to persistent pain and limited movements of the right shoulder. On inspection and examination of the right shoulder, swelling, together with tenderness to palpation were detected, so that a diagnosis of right shoulder effusion was made. Diclofenac and betamethasone were administered, and the patient was discharged home with an orthopedic consultation scheduled for the following day.

The next day, the patient returned to the first hospital. During the orthopedic examination, she reported experiencing fever with nausea and vomiting in the weeks prior, in addition to shoulder pain. An intra-articular injection of methylprednisolone was administered, before discharging the patient again.

On the same day, the woman’s general practitioner prescribed blood and urinary tests as well as an abdominal ultrasound due to a suspicion of gastroenteritis.

Later that day (four days after the first ED admittance), the emergency services were alerted by the patient’s husband after a syncopal episode. Upon the arrival of the ambulance, the woman was found in cardiovascular arrest. After 10 min of cardiopulmonary resuscitation, she was pronounced dead.

A forensic autopsy was requested by the judicial authority due to suspected medical malpractice, following a complaint of poor health care from the family of the victim.

## Material and methods

### Postmortem examination and additional analyses

A forensic autopsy was performed at the Institute of Legal Medicine of the University of Bologna, 8 days after the woman’s death, including external and internal examination. During the autopsy, samples of fluids and tissues were collected for microbiological, biochemical, toxicological as well as for histopathological analyses.

As biochemical markers of a suspected sepsis, C reactive protein (Atellica® CH Wide Range C-Reactive Protein (wrCRP) assay, measuring interval: 0.5–156 mg/L), troponin I (Atellica® IM High‑Sensitivity Troponin I (TnIH) assay, measuring interval: 2.50–25,000.00 ng/L) and procalcitonin (Atellica® IM BRAHMS PCT assay, measuring interval: 0.03–50,000.00 ng/mL), were measured. Limit of Detection (LOD) and Limit of Quantitation (LOQ) were, respectively, 0.30 mg/L and 0.50 mg/L for C reactive protein, 1.60 ng/L and 2.50 ng/L for troponin I, and 0.03 ng/mL and 0.04 ng/mL for procalcitonin.

Toxicological analysis included a liquid chromatography tandem mass spectrometry screening and quantification method for the detection of the most common drugs of abuse, drugs and psychoactive substances. The analysis took place at the Laboratory of Forensic Toxicology of the University of Bologna, using an internally validated method for forensic purposes by Waters Corporation [13].

The collected samples of body tissues were fixed in 10% pH 7 buffered formalin. Bone tissue, taken from near the right shoulder, underwent decalcification prior to formalin fixation. Histological slides were stained with a standard Hematoxylin and Eosin, as well as with additional stainings, particularly Grocott, Mallory’s P. trichrome, Perls, Periodic acid-Schiff (PAS), and PAS with Diastase (PAS-D). Immunohistochemistry for CD3, CD15, CD20 and CD68 were also performed. The histological slides were observed under a light microscope with magnifications ranging from 4 × to 40x. The shoulder radiological images, corresponding to the X-rays carried out at the ED, were requested to the hospital and anew analyzed with the assistance of a specialized radiologist in the setting of the post-mortem evaluation of medical liability.

### Literature review

A systematic review of the literature was conducted using “PubMed”, ‘Scopus” and ‘Web of Science” as medical databases. The key words used were: [(Autopsy) OR (Fatal) OR (Post-mortem) AND (Septic arthritis)], and [(Fatal septic arthritis)]. Inclusion criteria were: articles written in English and articles about fatal cases of septic arthritis. Cases involving in-hospital diagnosis followed by death were included. No time limits nor other filters were applied. The first selection was made by reading the titles of the articles, then another selection was based on the abstracts reading; lastly the whole text was read. The research was conducted from September 2023 until February 2024.

## Results

### Autopsy

**Fig. 1:**
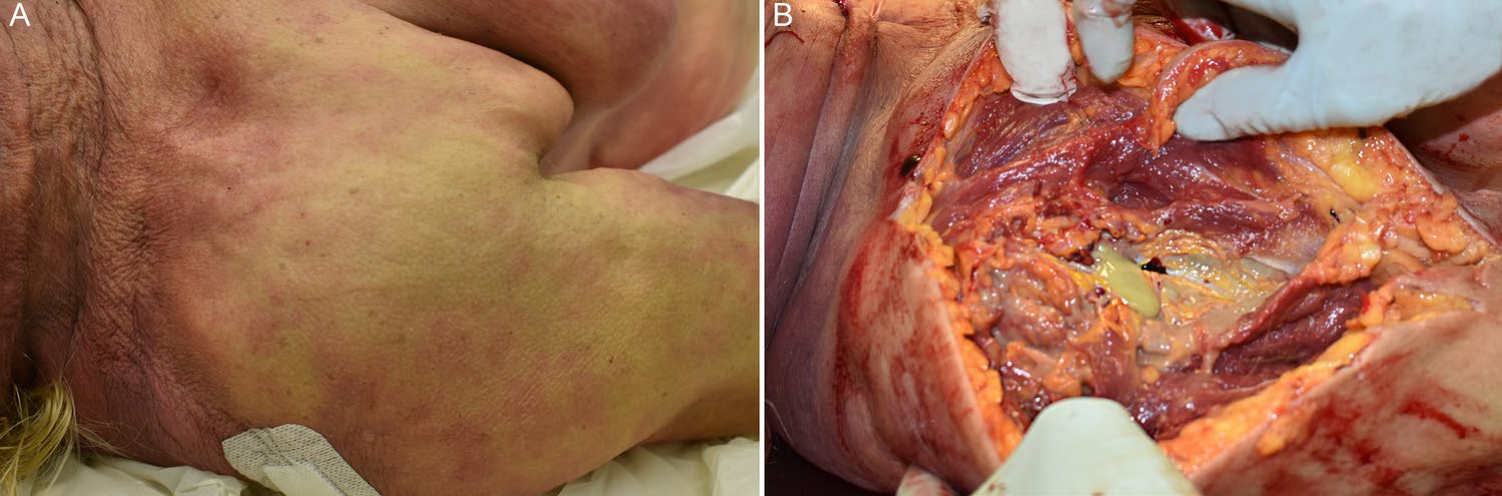
Right glenohumeral joint as shown at external examination (**A**) and after opening (**B**), showing purulent material within the articular capsule

The woman was overweight with a calculated BMI of 32. External examination was unremarkable with no signs of recent or previous injuries. The internal examination revealed multiple pleural and pericardial petechiae and polyvisceral congestion. The heart was enlarged and increased in weight (550 g), but in the absence of acute ischemic changes or other significant abnormalities. The coronary arteries were patent with moderate atherosclerosis but non-hemodynamically significant stenosis. Pulmonary edema was detected, with lungs weighing the right 720 g and the left 630 g, respectively. The brain was mildly edematous (1360 g). As no evident cause of death was found, the area of the right shoulder was dissected, in order to seek for changes in the corresponding glenohumeral joint. Upon incision, an abundant purulent yellow-greenish exudate filled the whole joint, spreading to the periarticular region covering, but not infiltrating, the surrounding muscles (Fig. 1). A microbiological swab of the exudate was taken, and a portion of the humeral bone together with the periarticular soft tissues was sampled and fixed in formalin for further histological analyses.

### Microbiology, biochemical and toxicological analysis

*P. multocida* was isolated from the swab taken during autopsy within the right glenohumeral joint.

Post-mortem biochemical analysis conducted on femoral blood provided the following results:

- C-reactive protein (CRP): 147.7 mg/L (reference values for adults: 0.00–5.00 mg/L);
- troponin I (TnIH): 16,567.70 ng/L (reference values for women: < 38 ng/L);
- procalcitonin (PCT): 0.09 ng/mL (reference values: < 0.5 ng/mL).

Toxicological analysis on femoral blood detected codeine (57.3 ng/mL), lidocaine and acetaminophen (not quantified but confirmed after screening).

### Histopathology

Microscopically, the examination of the joint showed marked chronic synovitis with few granulocytes (sub-acute/chronic synovitis). The synovitis was graded according to Krenn [14].

**Fig. 2:**
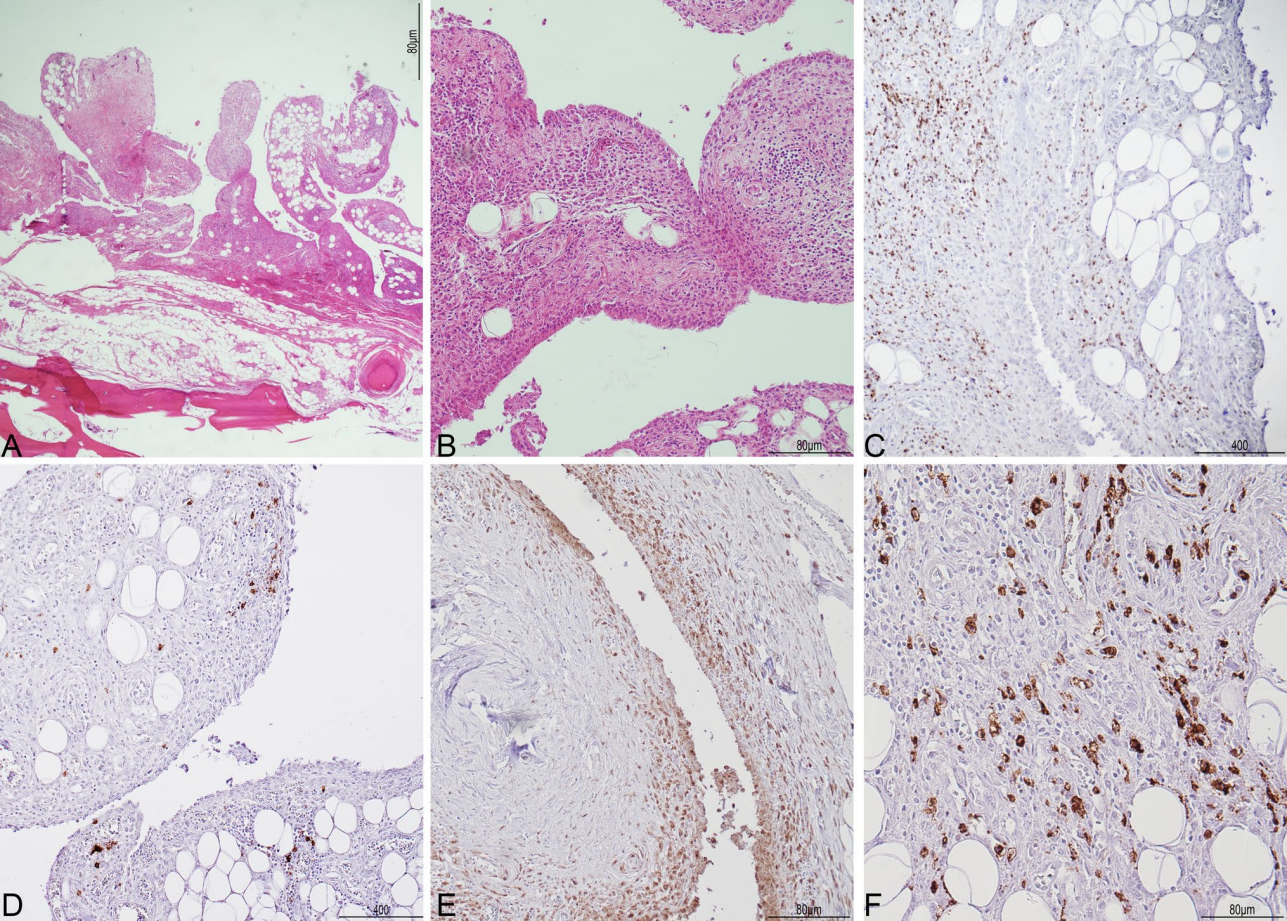
Histology of the glenohumeral joint. In A: the synovia (top), lying on the periosteum and bone (bottom), showed increased cellular proliferation with formation of small papillae (**A**, Hematoxylin and Eosin 2HPF). In B: chronic synovitis; a particular of the synovia with enlargement of the external cellular layer, abundant inflammatory infiltrate within a dense stroma composed of fibrosis, fibroblasts, and small vessels (**B**, Hematoxylin and Eosin 10HPF). Immunohistochemical characterization of the inflammatory infiltrate (**C**-**F**): CD3 T-lymphocytes were abundant (**C**), mixed with few CD20 B-lymphocytes (**D**); the histiocytes, positive for CD68-PGM1, were mainly located underneath the external cellular layer (E); scattered neutrophils, marked by CD15, were also observed (**F**)

The synovial lining cell layer was enlarged forming 2–3 layers (1 point) and in the stroma the cellular density was increased with fibrosis, fibroblasts, and vascularization (2 points). The inflammatory infiltrate was prominent with abundant lymphocytes (2 points). The final score was 5/9, indicating a high-grade synovitis (Fig. 2). Immunohistochemistry showed many CD3 + T-lymphocytes, some CD20 + B-lymphocytes, many CD68-PGM1 + histiocytes, and scattered CD15 + neutrophils, mainly found in the small vessels (Fig. 2).

The histochemical stainings for PAS, PAS-D, Grocott, and Pearl’s were negative.

**Fig. 3:**
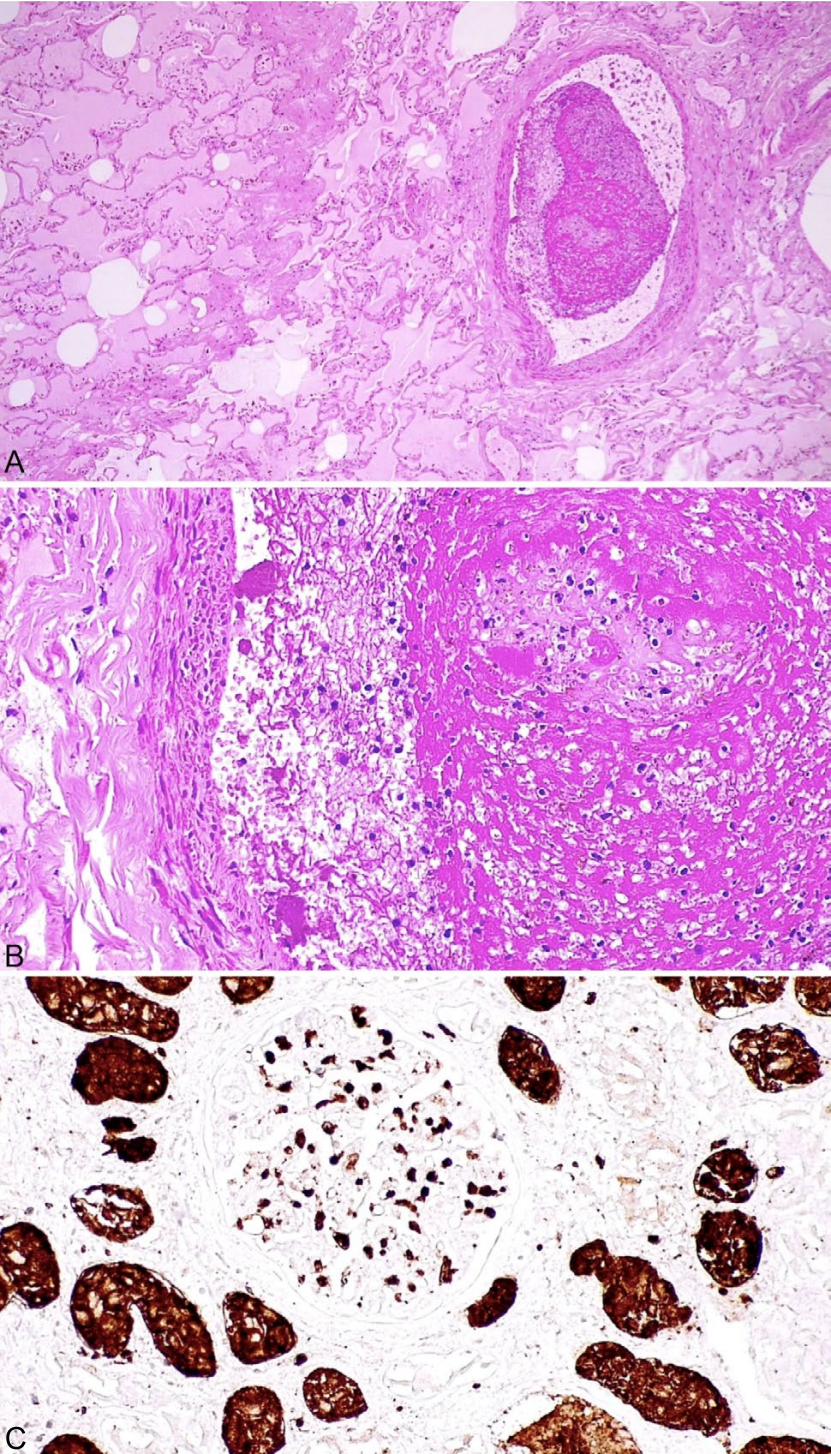
Right lung, superior lobe: diffuse pulmonary edema; a recent non-occlusive thrombus was observed in a middle size artery (**A**). It was composed of fibrin, many neutrophils and few macrophages, favouring a recent formation (**B**). In C, immunohistochemistry for CD15: numerous granulocytes (CD15 +) were present within the glomerular capillary network. CD15 was physiologically expressed in the tubular epithelial cells

Both lungs showed diffuse pulmonary edema. In the right lung, few non-occlusive thrombi were found in the peripheral branches of the pulmonary artery. The thrombi were composed of abundant neutrophils (CD15 +) and scattered macrophages (CD68-PGM1 +), embedded in fibrin, indicating a recent formation (Fig. 3). The histochemical stainings for PAS, PAS-D, Grocott, and Pearl’s were negative. The kidneys showed many CD15 + granulocytes within the glomeruli (Fig. 3). The histochemical stainings for PAS, PAS-D, Grocott, and Picro-Mallory were negative.

### Expert re-evaluation of the X-rays images

**Fig. 4:**
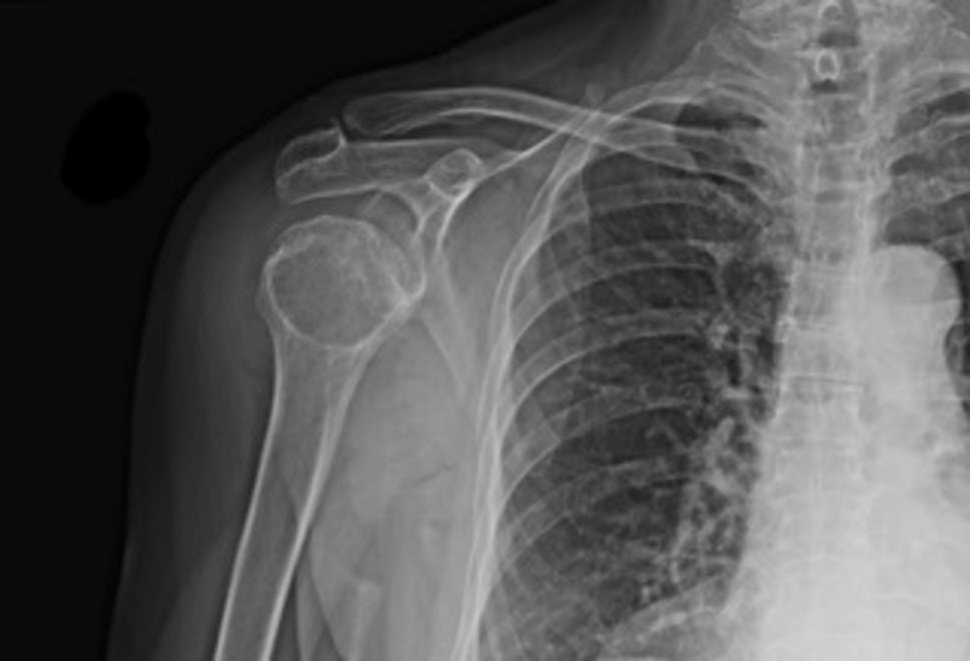
Radiological image of the right shoulder, showing periarticular soft tissue thickening and increased sub-acromion-deltoid space

The re-evaluation of the radiological images confirmed the arthrosis of the glenohumeral joint, as correctly reported at the ED. The analysis performed after the results of the autopsy also allowed the identification of periarticular soft tissue thickening and increased sub-acromion-deltoid space (Fig. 4).

### Additional information requested to the family

Given the microbiological isolation of *P. multocida*, upon authorization of the Judicial Authority more detailed information was requested to the patient’s family, regarding the occupational history, the possession of domestic animals, and previous traumatic injuries which might have been overlooked.

The family reported that the woman had been retired for several years and ruled out previous even mild traumas. The family also denied having any domestic animal, so that the detection of *P. multocida* remained unexplained. After a multidisciplinary re-evaluation of the postmortem data, the family of the deceased was again asked if the woman had any contact with animals, even of the wild type, especially cats. Only after more specific and reiterated questions to the family of the victim it was discovered that, a few weeks before death, the woman had rescued a wounded stray cat, from which she had been bitten and scratched.

### Literature review

A total of 607 articles were found after the first research on the medical databases. After skimming the titles and abstracts and applying the inclusion criteria, 16 articles were initially included. After reading the whole text of the selected papers and checking the references, 3 articles were excluded and 2 articles were added from the references. Lastly, 15 articles, published between 1980 and 2023, were considered in this review.

Out of 15 papers, 14 articles were case reports, only one was a retrospective study, for a total of 18 retrieved cases. The deceased were men in 10 cases (55.6%), women in 5 (27.8%), and the gender was not specified in 3 cases (16.6%). The reported ages ranged from 9 to 85 years old, with a mean of 55.7 year (SD 20.8) and a median of 53.5. All but one of the subjects had a positive medical history for: liver disease, diabetes, cancer, immunodepression, immune diseases, renal transplant, gout, joint prosthesis or other chronic diseases. For 17 of 18 patients (94.4%), at least one risk factor for the development of the specific infection was reported in the medical history, including a history of animal contact, a trauma or ingestion of seafood. All patients were hospitalized and received the diagnosis while still alive, mostly in association with blood or synovial cultures. The autopsy was performed in 6 cases (33.3%) and not performed in 7 cases (38.9%); in the remaining 5 cases (27.8%) there was no indication as to whether an autopsy had been conducted. *P. multocida* was only reported in one case. Other described pathogens included *Vibrio vulnificus*, *Staphylococcus aureus* and *lugdunensis*, *Aspergillus fumigatus*, *Mycoplasma hominis*, *Ureaplasma parvum*, *Escherichia coli*, *Mycobacterium abscessus*, *Burkholderia pseudomallei*, *Trichosporon beigelii*, *Corynebacterium striatum*, *Candida albicans*. Out of 18 cases, 10 were monoarticular infections (55.6%), 4 polyarticular (22.2%), and 4 unspecified (22.2%). The most frequently involved joint was the knee, followed by the shoulder and the elbow. The most frequent cause of death was septic shock and multi organ failure (M.O.F.). In the only 6 cases in which the autopsy was performed, the main findings were the evidence of diffused or localized infections. The cause of death was respectively: intra-cranial abscess, gas gangrene and septicemia, acute respiratory and circulatory failure, M.O.F., generalized convulsions and coma, septic shock; in one case out of 6, the cause of death was not specified.

The complete results of the review are summarized in Table 1.

**Table 1:** Review results

Author	Article type and n. cases	Age	Sex	Medical history	Hospital admission	Diagnosis alive	Cultures	Pathogen	N. of joints
Gerster et al. [15], 1980	Case report, 1	44	M	Hodgkin’s disease stage IIIB A Acute myeloblastic leukemia	Yes	Yes	Right knee’s synovial fluid (positive)	*Candida albicans*	1
Yangco et al. [16], 1982	Case report, 1	64	M	Alcoholism Chronic pancreatitis Laennec’s cirrhosis Hepatic encephalopathy Diabetes mellitus	Yes	Yes	Blood samples (positive) Left shoulder’s synovial fluid (positive)	*Escherichia coli*	1
Gardella et al. [17], 1985	Case report, 1	26	M	Severe aplastic anemia treated with allogeneic bone marrow transplant and subsequent graft rejection	Yes	Yes	Knees’ synovial fluid (positive)	*Trichosporon beigelii*	2
Cassuto-Viguier et al. [18], 1995	Case report, 1	47	/	Polycystic kidney disease (hemodialyzed) Renal transplant (rejected) Immunosuppressed CMV infection	Yes	Yes	Right shoulder’s synovial fluid (positive) Abscess of the skull (positive)	*Aspergillus fumigatus*	1
Kragsbjerg et al. [19], 2000	Case report, 1	79	M	Hypertension Psoriasis Seropositive rheumatoid arthritis with general joint affection Severe osteoarthritis Total replacement of the left knee Total replacement of the right knee	Yes	Yes	Blood samples (positive) Knees’ synovial fluid (positive)	*Staphylococcus lugdunensis*	2
Johnson et al. [20], 2001	Case report, 1	59	M	Hypertension Diabetes mellitus Presumptive gout Tobacco use^a^ Alcohol use^b^	Yes	Yes	Blood samples (positive) Right wrist’s synovial fluid (positive)	*Vibrio vulnificus*	4
Boltin et al. [21], 2009	Case report, 1	80	F	Myasthenia gravis (previously treated with pyridostigmine, plasmapheresis and azathioprine) Symmetric arthritis with pitting edema (RS3PE, managed with low dose steroids)	Yes	Yes	Right shoulder’s synovial fluid (positive) Blood samples (positive)	*Corynebacterium striatum*	1
Kuo et al. [22], 2009	Case report, 1	9	F	β-thalassemia major (chronic hemosiderosis)	Yes	Yes	Blood samples (positive) Right knee’s synovial fluid (negative) Urine (negative) Faeces (negative)	*Vibrio vulnificus*	1
MacKenzie et al. [23], 2010	Case report, 1	54	M	Non-Hodgkin lymphoma Delayed wound healing and fistula formation after implantation of a total hip prosthesis	Yes	Yes	Left knee’s synovial fluid (negative) Swabs and biopsy from the non-healing wound of the right hip (positive) Biopsy of resected aortic aneurysm (positive) Blood samples (positive)	*Mycoplasma hominis, Ureaplasma parvum*	4
Rafai et al. [24], 2013	Case report, 1	35	-	-	Yes	Yes	Yes (positive)	*Panton-Valentine leukocidin producing methi-S Staphylococcus aureus (MSSA)*	1
Fukui et al. [25], 2015	Case report, 1	75	F	Dermatomyositis Interstitial lung disease Pulmonary hypertension Chronic kidney disease Raynaud’s phenomenon Pulmonary tuberculosis Prolonged treatment with corticosteroids	Yes	Yes	Blood samples (positive) Right elbow’s synovial fluid (positive) Sputum (negative) Pleural fluid (negative)	*Mycobacterium abscessus*	1
Emamifar et al. [26], 2015	Case report, 1	80	-	Multiple myeloma Immunodepression Rectum cancer ITP^c^ MGUS^d^	Yes	Yes	Blood samples (positive) Right elbow’s synovial fluid (positive)	*Vibrio vulnificus*	1
Wu et al. [27], 2021	Retrospective study, 4	1) 53 2) 46 3) 38 4) 80	M M M F	1) Diabetes mellitus 2) Diabetes mellitus 3) Diabetes mellitus 4) Diabetes mellitus	1) Yes 2) Yes 3) Yes 4) Yes	1) Yes 2) Yes 3) Yes 4) Yes	1) Blood samples (positive), unidentified pus or fluid (positive) 2) Blood samples (positive) 3) Blood samples (positive), joint synovial fluid (positive) 4) Blood samples (positive)	1) *Burkholderia pseudomallei* 2) *Burkholderia pseudomallei* 3) *Burkholderia pseudomallei* 4) *Burkholderia pseudomallei*	-
Thi Hong Nhi et al. [28], 2023	Case report, 1	49	M	Cirrhosis	Yes	Yes	Peripheral blood samples (positive) Right knee’s synovial fluid (positive)	*Pasteurella multocida*	1
Umemoto et al. [29], 2023	Case report, 1	85	F	Hypothyroidism Lumbar vertebral compression fracture	Yes	Yes	Blood samples (positive) Right shoulder’s synovial fluid (positive)	*Corynebacterium striatum*	1

Author	Joint	Clinical examination	Laboratory	Imaging	Cause of death	Time of death	Autopsy findings	Risk factors	
Gerster et al. [15], 1980	Right knee	1st admission: “*increasing temperature and signs of acute right knee arthritis*” 2nd admission: “*fever, swelling of the right knee joint, severe neutropenia, thrombocytopenia and numerous myeloblasts in the blood smear”*	-	1st admission: Knee X-ray: negative 2nd admission: Knee X-ray: patchy lucencies in the femoral epiphysis and a small in the internal tibial margin	-	One year after the first hospital admission	Diffuse leukemic infiltrates of lymphohematopoietic organs, disseminated visceral foci of fungal infection presenting as pseudotumoral nodules; no joint effusion at the right knee, but the articular capsule was covered by a thick fibrinous layer; cartilage erosions on the tibial plateau and on the internal femoral condyle. Positive Grocott's stain of the capsule as well as in the eroded zone of the tibial cartilage, bone atrophy	Compromise of immune function	
Yangco et al. [16], 1982	Left shoulder	BT: 37° C HR: 100 bpm BP: 90/60 mmHg RR: 24 breaths per minute Examination: “*lethargic, disoriented, minimal nuchal rigidity, mild epigastric tenderness; pain elicited on moving the left shoulder but no inflammation*”	Hb: 15 g/dL WBCs: 14.6 × 10^9^/L (89% neutrophils, 10% lymphocytes) PLTs: normal	Chest X-ray (1st day): “*emphysema of the short* (sic) *tissue of the left shoulder joint*” Chest X-ray (3rd day): “*emphysema of the left side of the chest wall and gas distending the capsule of the glenohumeral joint*”	Gas gangrene and septicemia	4 days after the last hospital admission	Myonecrosis of the chest wall and left shoulder, extensive necrosis of the left shoulder joint capsule. At the left shoulder joint capsule, gram positive bacilli morphologically compatible with Clostridia organisms	Intra-articular steroid administration, compromise of immune function	
Gardella et al. [17], 1985	Right and left knees	Fever Examination: “*painful swelling with erythema in both knees*”	-	-	M.O.F., generalized convulsions and coma	75 days post-transplant	Multiple fungal abscesses in the liver, both kidneys, thyroid gland, bone marrow, myocardium, brain, and synovial membrane of both knees. Cultures positive for *Trichosporon beigelii*. Bone marrow aplasia	Bone marrow transplant and graft rejection	
Cassuto-Viguier et al. [18], 1995	Right shoulder	Examination: “*shoulder osteoarthritis and dermo-hypodermal lesion of the thigh*”. Few months later: *dysphonia and dysphagia. Neurological examination consistent with Collet-Sicard syndrome*	-	Increased technetium uptake at the right shoulder Few months later: MRI of the cranium showed a tumoral lesion of the skull base and prebulbar extradural empyema	Intra-cranial abscess	Few days after the last hospital admission	Abscess of the skull base	Compromise of immune function	
Kragsbjerg et al. [19], 2000	Right knee Left knee	In May 1995 “*bacterial arthritis of the right knee*” In June 1996 “*swelling, pain, effusion and warmth of the left prosthetic knee*”	From June 1996, when symptoms were present: CRP: 4–8 mg/dL ESR: 50–100 mm/hr	Left knee X-ray: signs of osteitis MRI: signs of vertebral osteomyelitis TEE: vegetation on the mitral valve (2 previous TEE were negative) TEE: vegetations on both the aortic and mitral valves	Acute respiratory and circulatory failure	2 years after the first hospital admission	Pneumonia, vertebral osteomyelitis, mitral and aortic valves infective endocarditis with vegetations and valves perforation	Treatment with corticosteroids and methotrexate Severe osteoarthritis with total replacement of the left knee	
Johnson et al. [20], 2001	Right wrist Right elbow Left knee Left lateral malleolus	BT: 38.9° C HR: 104 bpm BP: 113/62 mmHg RR: 22 breaths per minute Examination: lethargic, mildly icteric sclerae, dry oral mucosa, weak apical cardiac impulse, hepatomegaly, cool extremities; right wrist and left malleolus warm, swollen with overlying bulla, erythematous, and tender to palpation	WBCs: 6.4 × 10^9^/L (85% neutrophils) PLTs: 47 × 10^9^/L WBCs in the synovial fluid (right wrist): 62.2 × 10^9^/L, 96% neutrophils	No	Septic shock	Fourteen days after hospital admission	Not performed	Consumption of seafood	
Boltin et al. [21], 2009	Right shoulder	1st admission: “*weight loss, malaise and a tender, fluctuant mass in the right axilla, afebrile*” 2nd admission: “*fever and right shoulder pain*”	1st admission Hb: 9 g/dL CRP: 0.023 mg/dL ESR: 10 mm/hr	Chest CT: collection in the right shoulder joint space with no axillary mass, and no lymphadenopathy Abdominal CT: infiltration of the mesenteric fat 2nd admission: TEE (negative)	Septic complications (urinary tract infection, pseudomonal line sepsis and *Clostridium difficile* associated diarrhea)	Around 6 weeks after first hospital admission	-	Compromise of immune function	
Kuo et al. [22], 2009	Right knee	BT: 38.5° C HR: 140 bpm RR: 28 breaths per minute BP: 64/34 mmHg Examination: cramping abdominal pain, nausea, diarrhea for 2 days, lethargic, ill looking, febrile; right knee tender, erythematous and swollen	Hb: 6.4 g/dL WBCs: 8.6 × 10^9^/L PLTs: 183 × 10^9^/L CRP: 90.6 mg/dL Right knee’s synovial fluid was thick and purulent. Analysis of the aspirate showed WBCs count of 10.5 × 10^9^/L	Right knee MRI: “*necrotizing fasciitis and septic arthritis*”	Septic shock and M.O.F	Three days after hospital admission	Not performed	Ingestion of seafood 3 days before the illness	
MacKenzie et al. [23], 2010	Right knee Left knee Right hip Left hip	-	Hb: 8.6 g/dL WBCs: 6.9 × 10^9^/L CRP: 37.4 mg/dL	-	Septic shock	2 months after the first hospital admission	Not performed	Hip surgery	
Rafai et al. [24], 2013	Left knee	Fever BP: 110/60 mmHg RR: 30 breaths per minute SpO_2_: 94% Examination: left knee swollen, edematous with redness and pustules, impaired flexion	WBCs: 15 × 10^9^/L Synovial fluid: “*cloudy yellow liquid containing 100.000 cells/mm*^*3*^* predominantly neutrophils*”	Knee X-ray: negative Chest X-ray: “*bilateral interstitial infiltrates of the alveoli*” Chest CT scan: pulmonary embolism	Septic shock and M.O.F	36–72 h after hospital admission	Not performed	Previous knee trauma	
Fukui et al. [25], 2015	Right elbow	Right elbow and wrist swelling for a month; afebrile; right elbow, forearm and wrist joint swollen with tenderness, right arm with erythema and warmth	WBCs: 9 × 10^9^/L (neutrophils 77%, lymphocytes 20%) CRP: 2.6 mg/dL	Right elbow X-ray: osteolysis and pathological fracture of the right olecranon MRI: fluid in the right elbow joint and forearm, high-intensity lesions in the right olecranon which suggested an osteomyelitis	Respiratory failure due to disseminated candidiasis	Around 6 weeks after hospital admission	Not performed	Compromise of immune function, previous trauma	
Emamifar et al. [26], 2015	Right elbow	BT: 36.7° C HR: 78 bpm BP: 117/61 mmHg RR: 18 breaths per minute Examination: acute, severe pain in the right elbow and leg; 30° limitation of extension of the elbow, redness and swelling	Hb: 11 g/dL WBCs: 7.7 × 10^9^/L PLTs: 113 × 10^9^/L CRP: 11.7 mg/dL Right elbow joint synovial fluid: elevated levels of nucleated cells	Not performed	Septic shock	10 h after hospital admission and 20 h after symptoms onset	Not performed	Compromise of immune function	
Wu et al. [27], 2021	-	1) Pain in joints, febrile (> 38° C), chest pain and tightness, redness of surrounding skin, localized swelling 2) Pain in joints, redness of surrounding skin, localized swelling, shortness of breath 3) Pain in joints, restricted movement, febrile (> 38° C), redness of surrounding skin, localized swelling, fatigue, anorexia, headache or dizziness 4) Pain in joints, restricted movement, febrile (> 38° C), redness of surrounding skin, cough, localized swelling, diarrhea, anorexia	-	-	-	-	-	1–4) Compromise of immune function	
Thi Hong Nhi et al. [28], 2023	Right knee	BT: 36.6° C HR: 140 bpm BP: 116/60 mmHg SpO_2_: 100% with oxygen supply via mask (15 L/min) Examination: lethargic; hepatosplenomegaly; “*The right knee and surrounding areas were puffed out, and redness with some broken blisters*”	WBCs: 6.7 × 10^9^/L, 85.7% neutrophil PLTs: 26 × 10^9^/L CRP: 7.7 mg/L PCT: 10 ng/mL	U.S.: “*fluid in the right knee and inflammatory signs in surrounding tissues*”	Septic shock and M.O.F	3 days after hospital admission	Not performed	Unclear history of animal contact; compromise of liver function	
Umemoto et al. [29], 2023	Right shoulder	BT: 37.5° C HR: 97 bpm BP: 106/66 mmHg RR: 20 breaths per minute Examination: “*10-day history of fever and right shoulder pain*”*,* conjunctival petechiae; right shoulder joint swollen and with difficulties during movement	Hb: 10.3 g/dL WBCs: 7.1 × 10^9^/L (neutrophils 85,5%, lymphocytes 6%) PLTs: 110 × 10^9^/L CRP: 11.9 mg/dL	TTE: negative TEE: vegetation in the right aortic coronary cusp CT of the right shoulder: fluid collection at the shoulder joint	Septic shock	On day 84 after hospital admission	“*Perforation of the aortic left coronary cusp with vegetation; neutrophil infiltration into the aortic valve without the presence of bacteria*”	-	

*BT* Body Temperature, *HR* Heart Rate, *BP* Blood Pressure, *RR* Respiratory Rate, *TTE* Transthoracic Echocardiography, *TEE* Transesophageal Echocardiography, *WBCs* White Blood Cells, *Hb* Hemoglobin, *PLTs* Platelets, *CRP* C-Reactive Protein, *PCT* Procalcitonin, *ESR* Erythrocyte Sedimentation Rate, *M.O.F.* Multi Organ Failure

aTwo packs per day

b10-15 g/day

cIdiopathic Thrombocytopenic Purpura

dMonoclonal Gammopathy of Undetermined Significance-IgA type

## Discussion

*P. multocida* infection can produce a spectrum of human diseases, mostly corresponding to soft tissue infections, but it might also be seriously invasive [30]. Indeed, respiratory, bone and joint infections (septic arthritis) are reported. Generally, septic arthritis of a native joint can occur by hematogenous spread as well as direct inoculation of the joint following trauma, surgery or intra-articular injection, or continuous extension of a bone infection [31]. Most cases of septic arthritis by *P. multocida* occur after a cat or dog bite or scratch distal to the involved joint, without direct penetrating injury. However, approximately one-third of cases are not preceded by an animal injury. Usually, skin and soft tissue infections have a good outcome, especially in patients without significant risk factors, but mortality rates from *P. multocida* can range from 9 to 31%, without a prompt and correct diagnosis and treatment [32, 33].

In the case here described the challenge for forensic pathologists consisted in the absence of a clinical diagnosis for septic arthritis that, as shown by our systematic reviews, usually precedes the fatal outcome. Indeed, septic arthritis is not typically included among the primary substrates responsible for an unexplained death. In the case here reported, given the reported shoulder pain and the absence of any other pathological cause of death, the right shoulder was sectioned, leading to the detection of abundant purulent material and giving an input to more detailed examinations, particularly to the microbiological culture.

The isolation of *P. multocida* from the swab taken on the articular purulent material was initially considered with caution because of the time elapsed between the patient death and the sample collection (8 days), which might have led to a bacterial putrefactive growth or to postmortem bacterial transmigration. Bacterial growth may correspond to a true positive result in post-mortem samples (pure growth of a specific pathogen colonizing an otherwise sterile organ or fluid), but translocation (bacterial migration from the mucosal surface into the blood and internal organs after death) or contamination (incidental introduction of bacteria into the samples when they are obtained using non-sterile tools or operating in non-sterile environments) may also occur [34–37]. As *P. multocida* is neither a commensal microbe of human beings nor typical of post-mortem proliferation [38], and relying on the monomicrobial growth of a typical pathogenic microorganism on an isolated body area, as well as on the body storage at 4 °C, the bacterial growth was considered reliable. On the other hand, a blood culture was not performed due to the multiple possible biases arising from it.

Once detected the growth of the potential pathogen, *P. multocida*, a history of animal contacts was searched but, at first, not confirmed. Only after extensive questioning of the family, it was learned belatedly that the woman had been bitten and scratched by a stray cat.

The first symptoms of soft tissues infections caused by *P. multocida* usually arise within 24 h from the injury, and other manifestations (edema, swelling cellulitis) follow within 24–48 h [30]. However, the time required for a septic arthritis to develop is not fully defined in the current literature. Weber et al. [30] described 12 cases of patients with septic arthritis caused by *P. multocida*, in whom the condition was “long lasting” and difficult to diagnose “before a few weeks” after the first injection of the pathogen in the subcutaneous tissues [30].

In our case, the timeframe between the reconstructed exposition to *P. multocida* and the development of the arthritis was estimated as two to four weeks, consistent both with the prolonged bacterial incubation and the histopathological findings of sub-acute/chronic arthritis. Moreover, at autopsy, no evidence of residual cat bites and scratches were observed, but they might have been healed during the incubation period.

A further complication was represented by the paucity of ante-mortem clinical data. Indeed, when the patient was still alive, the right shoulder pain was attributed to degenerative polyarthritis, as confirmed by X-rays. The woman did not report of recent fever, which, as demonstrated in our review [28], might be absent in septic arthritis; vital signs abnormalities were not detected, and the condition was not further investigated by laboratory testing or ultrasound.

The re-evaluation of the radiological images contributed, however, to the comprehension of the case. Previous case reports of septic arthritis [39, 40] have shown aspecific signs at radiography, such as depression of the humeral head or widening of the sub-acromial space. These signs, additionally noted at the re-examination in our case, were interpreted as indicative of effusion and, therefore, inflammation, further confirming the suspect for septic arthritis.

The autopsy finding of septic arthritis, which typically involves a hematogenous spread of the pathogen, alerted for further analysis in order to establish whether a systemic infection and sepsis had occurred.

The diagnosis of sepsis is mainly clinical and requires multiple information and positive scorings performed in a hospital setting, which were unavailable in our patient [41].

As suggested by Stassi C. et al. [42], for the post-mortal diagnosis of sepsis it is advisable to collect all available data, including microbiological, biochemical analysis, and histopathological examinations, to make the most probable and accurate diagnosis.

Beside the above-mentioned microbiological analysis, the post-mortem biochemistry, conducted on femoral blood, oriented towards the diagnosis of sepsis, despite only two of the three measured markers were found above the reference values: CRP of 147.7 mg/L and TnIH of 16,567.70 ng/L, respectively. CRP is an acute-phase protein produced by the liver in response to inflammation and the elevated levels detected, compared with the literature, were consistent with sepsis prior to death. TnIH might reflect the myocardial damage associated with sepsis, but could be falsely elevated in heart blood in a post-mortem setting. In the present case, the biochemical analyses were conducted on femoral blood, so the values were considered indicative of the pre-mortem condition. PCT levels were within normal limits with 0.09 ng/mL (normal range < 0.5–2 ng/mL). Although PCT in septic arthritis can be of great value, as reported by a recent meta-analysis it cannot and should not be considered the only relevant marker for the diagnosis [43]. Moreover, PCT negativity does not necessarily contradict the hypothesis of septic arthritis, as post-mortem degradation of serum proteins and the low sensitivity (50–60%) must be taken into account [43].

Even if PCR [44–46] and troponin I values are not specifically indicative of a spreading bacterial infection, they were deemed highly suggestive of an ongoing inflammatory status at the time of death.

This systemic condition was also sustained by the observation of non-occlusive initial thrombi composed of fibrin and granulocytes within the small pulmonary vessels. These ancillary findings were consistent with the hypothesis of an infectious/septic state [47].

Granulocytes, immunohistochemically marked by CD15, were also found in the renal glomeruli, indicating an ongoing chemotactic process [48, 49].

On this basis, the cause of death was deemed as a *P. multocida* septic arthritis with likely evolution into sepsis and septic shock. The deceased was previous healthy, except for a moderate osteoarthritis.

Despite the absence of an ante-mortem diagnosis and although several tests were not performed at the ED (e.g. blood analysis and ultrasonography of the shoulder), the suspicion for medical liability was waived as the correct diagnosis in life would have been challenging (clinical signs, symptoms and radiological images were non-specific and the contact with a stray cat was not medically reported) and the mortality of septic arthritis is reportedly high. On the other hand, the local infiltration of methylprednisolone might have facilitated the proliferation of *P. multocida* in the joint cavity as well as its spreading, by inhibiting the migration and phagocytic function of neutrophils and macrophages.

Our literature review further highlighted that septic arthritis as a cause of death is rarely encountered in forensic pathology, because the diagnosis is usually established ante-mortem. Moreover, other pathogens than *P. multocida* are usually involved. Indeed, only one other case of fatal septic arthritis due to *P. multocida* was reported and the pathogen was identified during ante-mortem cultures. However, as emerged in the cases here revised, symptoms of septic arthritis might be non-specific (with no fever, general malaise, signs of arthritis) leading to delayed or missed diagnosis [15, 17, 19, 21, 23, 25, 29]. In similar cases, a judicial autopsy might be requested to clarify the mechanism of death or for a suspicion for medical malpractice.

According to our results, fatal outcomes in septic arthritis are more frequently reported in middle-aged male patients, with associated conditions such as systemic or immune involvement, that should raise a high level of suspicion for infective causes of death in the forensic pathologist.

Individuals with defective immune system are particularly at risk for *P. multocida* infections, which can be additionally more severe in children or in older people [50, 51], underlyining the need for a greater vigilance in these subpopulations. Nevertheless, even healthy subjects may develop *P. multocida* widespread septicemia, sepsis, and septic shock, as shown for other pathogens such as *Corynebacterium striatum* [2, 21, 29, 52, 53].

Beside conditions compromising the liver or immune function, septic arthritis shows a predilection for affecting joints already damaged by trauma [24], surgery [23] or degenerative diseases, as degenerative osteoarthritis in the case here described [54–58]. Similar conditions, with particular reference to degenerative diseases, might be increasingly encountered in the forensic practice with the aging population, and it should be kept in mind that a concurrent infection might occur.

A clear history, e.g. of animal contacts, might not be reported, underlying the need for a greater awareness among doctors regarding the potential complications of animal-related injuries and the importance of a targeted history. Particularly, when preexisting degenerative diseases are considered responsible for the pain in the clinical setting, the autopsy might represent the only mean to achieve a diagnosis. In this setting, risk factors for septic arthritis should be documented during the post-mortem examination or sought for during the forensic investigation, including contacts with animals.

The first indication at autopsy, beside the absence of other pathological causes, might be represented by purulent material at the joint or abscesses and these findings should initiate the careful collection of microbiological samples. Additionally, signs of sepsis in other tissues such as thrombi in the kidneys or in the lungs should be sought, although most postmortem findings reported in the literature are non-specific.

In cases of suspected sepsis with limited pre-mortem information and an unclear focus of infection, the examination of joints during autopsies is recommended and may represent the only opportunity for a forensic diagnosis of the cause of death. This practice can uncover critical information that may have been overlooked during clinical evaluations and can also clarify infection pathways, address clinical discrepancies, and ultimately contribute to enhancing future patient care in septic conditions. The insights gained from such examinations can significantly impact clinical approaches to managing sepsis and related complications.

In conclusion, the autopsy performed in a multidisciplinary manner, corroborated by ancillary tests and thorough circumstantial data, still remains the gold standard examination in explaining the cause of death, despite the complexity of the case. Moreover, injuries provoked by domestic or wild animals should not be underestimated, even if they leave only mild or no sign, and a history of animal scratches and bites, if reported, should raise the suspicion for serious infections and even a septic death.

## Conclusion

We presented an unusual case of death resulting from *P. multocida* septic arthritis in a 67-year-old woman who had complained of pain to the right shoulder. To the best of our knowledge, the case presented is the first in which the diagnosis of septic arthritis due to *P. multocida* was made post-mortem. Given the challenge of the clinical diagnosis, the post-mortem examination, following a multidisciplinary approach including integration of the clinical history, re-evaluation of the imaging, dissection of specific sites at autopsy, microbiological and histopathological analysis, could represent the only opportunity for the diagnosis of the cause of death.

## Key points

1. Septic arthritis might represent an under-reported clinical and pathological diagnosis.
2. Deviations from the usual autoptic technique might be required to diagnose a septic arthritis.
3. The autopsy could prompt the collection of circumstantial data otherwise neglected.
4. The multidisciplinary approach is crucial in cases of suspected articular infection.

## Data Availability

Data are available by request to the corresponding author.
